# Partial proportional odds model for predicting multiple lower extremity amputation among T2DM patients

**DOI:** 10.1186/s12911-025-03112-6

**Published:** 2025-08-04

**Authors:** Sabiha Khan, Karuna Reddy, Momtaz Ahmed, Donald Wilson, Bibhya Sharma

**Affiliations:** 1 School of Information Technology, Engineering, Mathematics & Physics, The University of the South Pacific, Suva, Fiji; 2 College of Medicine, Nursing and Health Science, Fiji National University, Suva, Fiji; 3 Diabetic Hub, Colonial War Memorial Hospital, Suva, Fiji

**Keywords:** Lower extremity amputation, Multiple-amputation, Risk factors, Statistical model, Ordinal logistics regression, Partial proportional odds model, Multinomial logistic regression

## Abstract

**Background or introduction:**

Multiple Lower extremity amputation (MLEA) is an unfortunate outcome following a lower extremity amputation (LEA) in individuals with diabetes. The challenges faced by individual with MLEA are significantly higher than those who have undergone a single amputation. Therefore, developing a reliable and accurate method for determining risk factors associated with MLEA is essential for reducing the incidence of this outcome among diabetic patients.

**Objectives:**

This study aimed to explore the demographic and clinical characteristics of diabetic inpatients with foot ulcers. The goal was to develop a statistical model to determine the risk factors of MLEA among patients type 2 diabetic mellitus (T2DM).

**Methods:**

Data for statistical model development were collected from patients’ folders involving 1,972 patients with T2DM who were hospitalized for acute diabetic foot ulcers (DFU) at three tertiary care hospitals in Fiji from 2016 to 2019. This cross-sectional study was conducted in accordance with the STROBE guidelines focusing on patients who experienced MLEA at the hospitals. Patients were categorized into three ordinal outcomes: no-amputation, primary amputation, and multiple amputations. A partial proportional odds model was developed to fit the ordinal outcome and determine the risk factors associated with MLEA. The proposed model was validated by comparing it to a proportional odds model and a multinomial logistics regression model.

**Results:**

The proposed partial proportional odds model (PPOM) identified several risk factors for MLEA, including age, gender, ethnicity, hypertension, anemia, leukocytosis, and thrombocytosis.

**Conclusions:**

The analytical findings reveal that the PPOM is appropriate for determining the risk factors associated with MLEA in T2DM patients in Fiji.

**Supplementary Information:**

The online version contains supplementary material available at 10.1186/s12911-025-03112-6.

## Introduction

The International Diabetes Federation (IDF) reports there were 537 million adults living with diabetes worldwide in 2021. This figure is continuously growing and is expected to rise to 783 million by year 2045 [1]. Moreover, type 2 diabetes mellitus (T2DM) is the most common form of diabetes. The global incidence of T2DM and its associated complications continue to rise. By 2030, projections indicate that the prevalence of T2DM could rise approximately to 4.4% worldwide [2–4].

The incidence of T2DM and its related complications is also increasing in the Pacific Islands region. It is estimated that nearly 1 in 3 adults in the Pacific population has T2DM [5]. Fiji is not exempt from this issue; one in every three Fijians are being diagnosed with diabetes, accounting for 30% of the general population. T2DM is more prevalent than any other type of diabetes in the country. In fact, Fiji has one of the highest diabetes rates in the world, with a prevalence of 16%, which is twice the global average of 8.5% [6].

Multiple Lower extremity amputation (MLEA) is an unfortunate outcome following a lower extremity amputation (LEA) episode in individuals with T2DM [7]. It was defined as those who required a new amputation or re-amputation at a proximal level in the same extremity or at the same or more proximal level in the opposite extremity [8–12]. The incidence of diabetic peripheral neuropathy and peripheral arterial disease is a major contributor to LEA [13]. It is usually performed to manage infections; however, infections often persists, necessitating MLEA [14]. MLEA may also be required for patient who have previously undergone LEA or experienced ulceration in either foot [8, 14–18].

Despite the high prevalence of T2DM in the Pacific region including Fiji, there is a lack of current statistics on LEA and MLEA, as well as their associated risk factors. The objective of this study is to identify the associated risk factors for MLEA in T2DM patients and develop statistical models to accurately determine these risk factors.

## Materials and methods

This study examines the demographic and the clinical characteristics of the T2DM inpatients to identify the potential risk factors for MLEA. Since MLEA was ordinal variable, we propose using a partial proportional odds model to predict risk factors of MLEA among T2DM patients. Additionally, we developed a proportional odds model (POM) and a multinomial logistics regression model (MLR) for predicting MLEA to compare the performance of the proposed model.

The performance of the proposed models developed was compared using several statistical measures, including the likelihood ratio test (LRT), Akaike information criteria (AIC), and Bayesian information criteria (BIC). The empirical results reveal that the proposed partial proportional odds model outperformed the other models, proving to be more efficient and accurate in predicting MLEA compared to the POM and MLR.

### Data and variable

This is a retrospective cross-sectional study and the data were collected from the medical records of all T2DM patients hospitalized for acute foot ulcers requiring further management between January 1, 2016 and December 31, 2019, at three tertiary care hospitals in Fiji: Colonial War Memorial Hospital, Lautoka Hospital and Labasa Hospital. These hospitals serve as the primary centers for surgical care in the country, providing specialized treatment for all forms of diabetic foot diseases and receives referrals from all other health facilities across Fiji. This paper follows the Strengthening the Reporting of observational studies in Epidemiology (STROBE) guidelines for the reporting of observational research. Prior to data collection, ethical approval for this study was obtained from the appropriate ethic committee: College Human Health Research Ethics Committee (CHHREC), Fiji National University.

Fiji has an electronic database known as the Patient Information System (PATIS), which serves as a national repository linked to each patient’s unique National Health Number (NHN). PATIS contains detailed records on patient admissions and discharge dates, International Classification of Disease (ICD-10 AM) codes, and surgical procedure codes. For this study, all patients with T2DM who had foot ulcers were identified from the hospital discharge lists in PATIS. The ICD-10-AM and procedure codes were sourced directly from these discharge lists, assigned by clinical coders at time of discharge in accordance with Australian Coding Standards (ACS).

**Fig. 1:**
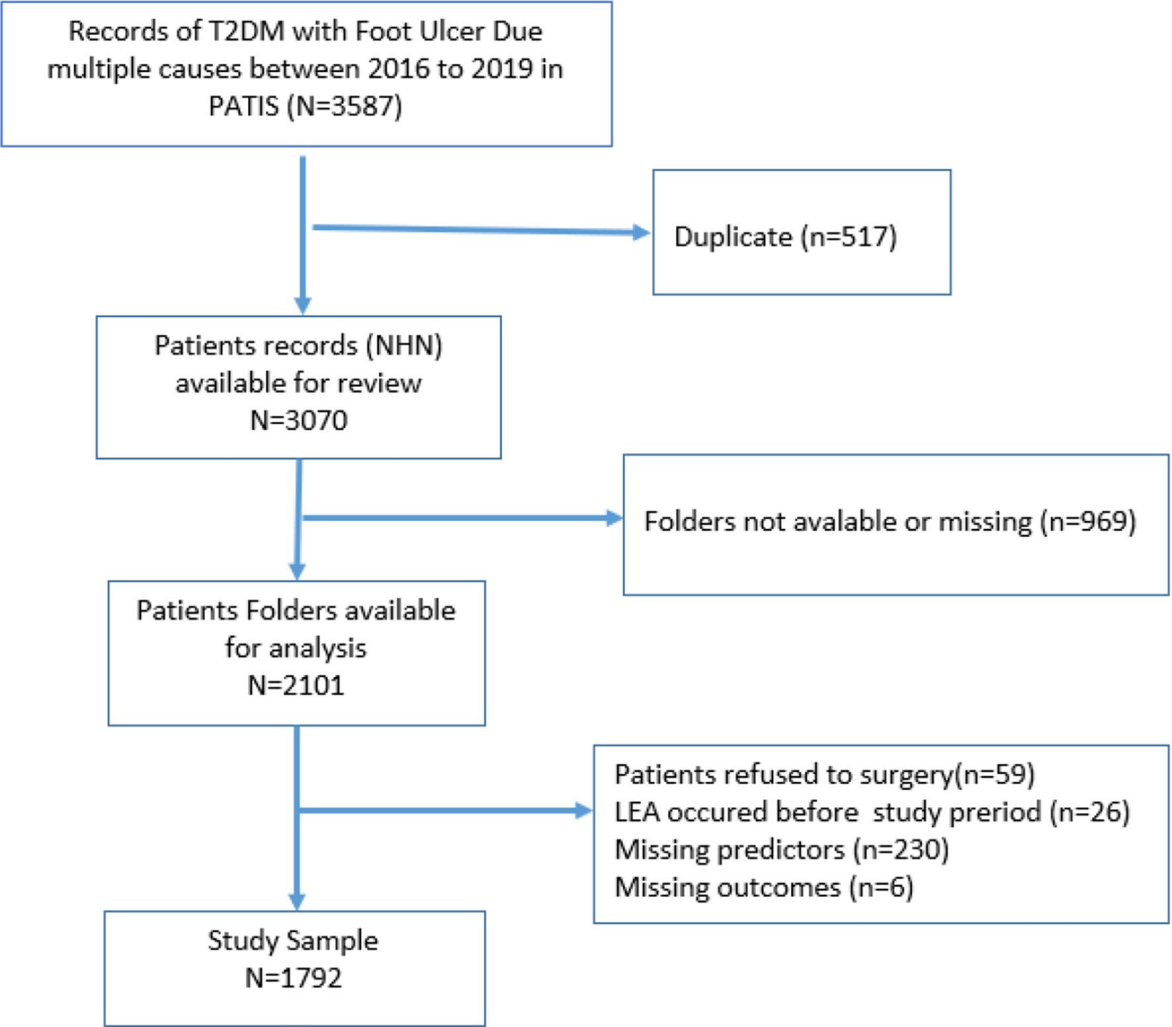
Flowchart of subject selection

Initially, a total of 3587 patients with T2DM and had foot ulcers were identified. After removing the duplicate records (*n* = 517), missing folders (*n* = 969), patients who refused surgery (*n* = 59), those who had undergone any LEA before the study period (*n* = 26), patients with incomplete information on any predictor variables (*n* = 224), and incomplete records on the history of LEA (*n* = 6), a total of 1792 T2DM patients with active foot ulcer were identified as study population who were admitted at three tertiary care hospitals in Fiji for further management. Figure 1 shows the flowchart of patients’ selection in the study.

The outcome variable was LEA status, which was categorized into three-category ordinal responses based on the number of amputation(s) experienced by a T2DM patient: No-LEA (NLEA), primary LEA (PLEA), and multiple LEA (MLEA). The LEA status was determined from post-operative notes at the time of discharge from a patient’s medical record. NLEA refers to patients who did not undergo amputation but had surgical interventions such as debridement or incision and drainage (I&D), PLEA refers to patients who had undergone one amputation defined as the surgical removal of bones and soft tissue at any level of the lower extremity for the first time during the study period, MLEA refers to patients who had multiple amputations defined as those who underwent more than one amputation during the study period. For patients who had multiple amputations on the same extremity or bilaterally within the same hospitalization, the most proximal level of amputation was considered, and earlier episodes were disregarded to prevent multiple counting. Each category was treated as a distinct level within an ordinal outcome variable. This ordinal categorization reflects the clinical progression in the severity of diabetic foot complication and limb loss. Ordinal regression modelling was employed to assess factors associated with increasing severity of LEA. This method is appropriate as it accounts for the ordered nature of the outcome variables, allowing estimation of the odds of being in a higher amputation category relative to lower categories, without combining or collapsing categories.

For each patient, all available complete data from their medical records routinely collected during their episode of care were retrieved as explanatory variables. This data included: demographic (age, sex, ethnicity, place of residence); lifestyle factors (smoking, alcohol), history and examination (duration of foot ulcer before admission, Wagner grades, body temperature, systolic blood pressure, diastolic blood pressure, admission date, and discharge date), co-morbidities (hypertension, anemia, Chronic kidney disease, ischemic heart disease, stroke, myocardial infarction, and coronary artery bypass grafting), investigations (uncontrolled blood sugar levels, leukocytosis, and thrombocytosis).

Considering the above variable in more details, a duration of ulcer exceeding one month prior to admission was defined as a late presentation, a body temperature of 38º C and above was considered as fever [19]. The length of hospital stays (los) was calculated from the admission date to discharge date. Hypertension was defined as a systolic blood pressures ≥ 140 mmHg, a diastolic blood pressures ≥ 90 mmHg, or evidence of the use of antihypertensive medications [19, 20], according to World Health Organization criteria, anemia was considered having a hemoglobin level of 12 g/dl for females and 13 g/dl for males. Chronic kidney disease (CKD) was defined as an estimated glomerular filtration rate (eGFR) of < 59 ml/min/1.73 m2, corresponding to stage 3a or worse [21]. The presence of ischemic heart disease (IHD), stroke, myocardial infarction (MI), or a history of coronary artery bypass grafting (CABG) was recorded if documented in the patient’s records. Random blood sugar (RBS) levels above 10 mmol/L were considered indicative of uncontrolled blood sugar [21]. Wagner Classification ulcer grading was determined from clinical notes in the patient’s records. Wagner Grade 0–3 is mainly based on neuropathy; with grade 4–5 representing ischemic lesions [22]. Presence of leukocytosis was considered if white blood cell (WBC) exceeded a count of 12,000 [23], and thrombocytosis was defined as a platelet count greater than 450,000 [24].

### Statistical analysis

In this study, both descriptive and inferential statistical analyses were used to analyze the data. Continuous variables were reported as mean ± standard deviation or median (interquartile range). Categorical variables were expressed as frequencies with percentages. One-way analysis of variance (ANOVA) or Kruskal–Wallis test (in case of non-normal data) was used to assess the relationships between the continuous predictor and the outcome variable. The Chi-square test or Fisher’s exact test was used to evaluate the differences between a categorical predictor and outcome variable. For all tests, a p-value < 0.05 was considered as statistically significant at 5% significance level.

Out of 20 available predictors, 19 variables were considered in the complete model. The Wagner grade variable was excluded from the complete model because it was observed that some levels of Wagner grade perfectly predicted the LEA categories, resulting in the problem of “perfect separation”. This resulted in model convergence failure, which resulted in large coefficient estimates and inflated standard errors, indicating infinite maximum likelihood estimates. Such separation violates the assumptions of the model and renders the effect estimates inconsistent and unreliable [25, 26]. Therefore, to ensure model stability and valid inference, we excluded Wagner grade from the model.

A stepwise ordinal logistic regression was used to select the final model. The complete and reduced models along with logit link was used to generate the final model. The complete model contained all the explanatory variables while the reduced model included a subset of some explanatory variables. Based on these results, 10 out of 19 variables remained in the final model which includes age, gender, ethnicity, illness duration, length of stay, hypertension, anemia, renal problem, leukocytosis, and thrombocytosis. Before reaching the final model, investigations surrounding multicollinearity among the explanatory variables indicated that no such problems were present in the data.

For each of the predictors, the odds ratios with their corresponding 95% confidence intervals were estimated to assess the strength of the association. The Brant test was used to assess the validity of the proportional odds regression model. Multicollinearity was also checked among the independent variables using the variance inflation factor (VIF) and with values above 10 indicating the presence of multicollinearity. All analysis was carried out using the R statistical software.

### Statistical models

For modeling ordinal categorical outcomes, the ordinal logistics regressions (OLR) models are commonly used [26]. Many OLR models that exist such as the proportional odds model (POM), the partial proportional odds model (PPOM), continuous ratio model (CRM), and stereotype model (SM) [26–31].

The POM is one of the most widely used logistic regression model for assessing ordinal outcome variable which is a member of the class of cumulative models [25, 26, 32–35]. However, it is strictly built based on the proportional odds or the parallelism assumption which means the effect of each predictor on the outcome is the same for all levels of the outcome [26, 36]. When the proportional odds (PO) assumption holds, the POM is considered as the most appropriate model for ordinal response variable [26, 37]. Violation of the proportional assumption, the POM becomes inappropriate as it can produce biased results and incorrect standard errors [37].

Although POM is suitable for analyzing ordinal outcome variables, the PO assumption is rarely hold in practice [36]. For a POM to be valid, the PO assumption must be tested, the Brant test is one method to assess this assumption. The Brant test compares the slope coefficients of (j − 1) binary logit implied by the ordered logistic regression model. It uses a series of Wald Chi-square tests for all predictor variables comparing different levels of response variable [38].

If the test shows that the assumption is violated for one or more variables in a model, remodeling the data with a newer strategy may be needed. The Partial Proportional Odds Model (PPOM) is the one way to remodel ordinal response data when the POM is inadequate due to violation of the PO assumption [36, 37, 39]. Previous studies have reported that PPOM is more appropriate than the POM when the PO assumption is violated [40, 41], as it allows for relaxation of this assumption for one or a small set of predictors [31, 36, 37, 39].

In this Study, to identify risk factors associated with MLEA, an ordinal logistic regression model (OLR) where the ordered categories of LEA were NLEA, PLEA, MLEA. In other words, if Y = number of amputation(s) experienced by a patient, then it is defined by Eq. (1) below:

1 $$Y=\left\{ \begin{gathered} {\text{0, NLEA,}} \hfill \\ 1,{\text{ PLEA}} \hfill \\ 2,{\text{ MLEA}}{\text{.}} \hfill \\ \end{gathered} \right.,$$

Although the Binary Logistic Regression has been performed by many authors, no such study considered the ordered outcome of LEA as an ordinal variable as mentioned Eq. (1) above. Furthermore, amongst the OLR models, POM and PPOM are most widely used in epidemiological and biological applications, our focus of this statistical inquiry is centered on the decision-making process of POM and PPOM.

Furthermore, we employed POM for modeling due to its interpretability, computational stability, and ability to yield cumulative odds ratios, which are particularly informative when assessing ordinal response categories. Although alternative models such as CRM and SM were considered, they were not pursued for the following reasons:

CRM: This model requires stronger distributional assumptions and does not provide cumulative odds ratios, limiting its practical interpretability in applications where ordered comparisons are essential [25].

SM: While flexible and suitable for relaxing the proportional odds assumption, SM estimates category-specific scores that may be difficult to interpret and are sensitive to sample size imbalances and category separation [25, 28].

For the comparison of models, we also use the multinomial logistic regression model (MLR), which is commonly used technique when the outcome variable is nominally categorized into more than two levels.

#### Proportional odds model

The proportional odds model (POM), also known as the ordinal logistic regression model described by McCullagh [26], is a type of regression model used for ordinal categorical outcomes. It provides a useful extension of the binary logistic regression model to handle ordinal response variables, where the response variable has more than two ordered categorical values [38].

Mathematically, the proportional odds model assumes that the odds of being in a higher category (or equal to) versus being in a lower category (for each category except the highest) are proportional across different levels of the predictor variables.

The proportional odds model can be expressed as in Eq. (2) [42]:

2 $$\log \left( {\frac{{P\left( {{Y_i} \leqslant k\left| {{x_i}} \right.} \right)}}{{P\left( {{Y_i}>k\left| {{x_i}} \right.} \right)}}} \right)={\alpha _k}+{\beta _1}{x_{i1}}+{\beta _2}{x_{i2}}+ \cdots +{\beta _p}{x_{ip}}$$

where:

${Y_i}$ = the ordinal response for the 𝑖th observation, which takes values from 1 to *J*, where *J* is the number of categories,

${x_{ij}}$ = the *j*th predictor for the *i*th observation, $j=1,2,...,p$

${\beta _j}$= the coefficient of the *j*th predictor,

$k=1,2,...,J - 1$ represents each of the lower $J - 1$ categories,

${\alpha _k}$= the intercept for the 𝑘th category, representing the log-odds of being in or below category 𝑘 versus being above category 𝑘,

*p* = the number of predictor variables.

Note that the description of the model given in Eq. (2) is perhaps counterintuitive in that high values of $\eta ={\alpha _k}+{\beta _1}{x_{i1}}+{\beta _2}{x_{i2}}+ \cdots +{\beta _p}{x_{ip}}$ are associated with low values of ${Y_i}$. For this reason, a preferred proportional odds model is given by Eq. (3):

3 $$\log \left( {\frac{{P\left( {{Y_i} \leqslant k\left| {{x_i}} \right.} \right)}}{{P\left( {{Y_i}>k\left| {{x_i}} \right.} \right)}}} \right)={\alpha _k} - {\beta _1}{x_{i1}} - {\beta _2}{x_{i2}} - \cdots - {\beta _p}{x_{ip}}$$

so that the sign of ${\beta _j}$ has usual meaning, that is, if positive, an increase in ${x_{ij}}$is associated with an increase in ${Y_i}$.

The Eq. (3) states that the log odds of being in or below category 𝑘 versus being above category 𝑘 is a linear function of the predictor variables. The coefficients ${\beta _j}$​ represent the effect of the predictor variables on the log odds, and ${\alpha _k}$ represents the baseline log odds for each category.

The “proportional odds” assumption implies that the effect of the predictor variables is consistent across the different category thresholds. In other words, the odds ratios comparing adjacent categories are assumed to be constant across all categories.

#### Partial proportional odds model

The partial proportional odds model (PPOM) is an extension of the proportional odds model (POM) that relaxes the assumption of proportional odds for some of the predictor variables while maintaining it for others. In the PPOM, some predictor variables are allowed to have effects that vary across different categories of the ordinal response variable, while other predictor variables have effects that remain proportional across categories [36].

Mathematically, a PPOM model can be defined as in Eq. (4) [36]:

4 $$\eqalign{\log \left( {{{P\left( {{Y_i}k\left| {{x_i}} \right.} \right)} \over {P\left( {{Y_i} > k\left| {{x_i}} \right.} \right)}}} \right) & = {\alpha _{1k}} - \sum\limits_{j = 1}^p {{\beta _{1j}}{x_{ij}}} - \cr& \sum\limits_{j = 1}^p {{\beta _{2j}}\left( {{x_{ij}} - {{\bar x}_j}} \right)};\quad k = 1,2, \ldots,J - 1 \cr} $$

where:

${Y_i}$ = the ordinal response for the 𝑖th observation, which takes values from 1 to *J*, where *J* is the number of categories,

${x_{ij}}$ = the *j*th predictor for the *i*th observation, $j=1,2,...,p$

${\beta _{1j}}$= the coefficient of the *j*th predictor variable for proportional odds part,

${\beta _{2j}}$= the coefficient of the *j*th predictor variable for non-proportional odds part,

${\alpha _{1k}}$= the intercept for the 𝑘th category of the proportional odds part, representing the log-odds of being in or below category 𝑘 versus being above category 𝑘,

*p* = the number of predictor variables,

${\bar {x}_j}$ = the mean of the *j*th predictor variable across all observations.

In this model, some predictor variables, those associated with ${\beta _{1j}}$, have effects that remain proportional across the different categories of the response variable, while other predictor variables, those associated with ${\beta _{2j}}$have effects that vary across categories. The term $\left( {{x_{ij}} - {{\bar {x}}_j}} \right)$ allows the effect of the predictor variable to vary around its mean. The intercepts ${\alpha _{1k}}$ are allowed to differ for each category of the response variable.

#### Multinomial logistic regression model

Multinomial logistic regression (MLR) is widely used model for predicting discrete outcomes when the depended variable is categorical with more than two levels, which are not necessarily ordered. It is an extension of logistic regression model to allow modeling of multiple categories [25, 43].

Mathematically, the multinomial logistic regression model is given by Eq. (5):

Let *Y* be a categorical dependent variable with *k* categories ($k=1,2,...,J$) and let *X* be a matrix of independent variables with *p* predictors. Then, the MLR model can be expressed as:

5 $$\eqalign{\log \left( {{{P\left( {Yk\left| X \right.} \right)} \over {P\left( {Y > J\left| X \right.} \right)}}} \right) & = {\beta _{0k}} + {\beta _{1k}}{X_1} + {\beta _{2k}}{X_2} \cr & + \cdots + {\beta _{pk}}{X_p};\quad k = 1,2, \ldots,J - 1 \cr} $$

Where:

$P\left( {Y \leqslant k\left| X \right.} \right)$ is the probability of the dependent variable *Y* being in category *k* given the values of the independent variables *X*.

$P\left( {Y \leqslant J\left| X \right.} \right)$ is the probability of the dependent variable *Y* being in the reference category *J*.

${\beta _{0k}},{\beta _{1k}}, \cdots,{\beta _{pk}}$= the coefficient of the *j*th predictor of category *k*,

${X_j}$= is the *j*th predictor, $j=1,2,...,p$

The parameters ${\beta _{0k}},{\beta _{1k}}, \cdots,{\beta _{pk}}$ are estimated using maximum likelihood estimation (MLE) or other optimization techniques to minimize the loss function, typically the negative log-likelihood function. The probabilities $P\left( {Y \leqslant k\left| X \right.} \right)$ are estimated using the softmax function:

$$P\left( {Y \leqslant k\left| X \right.} \right)=\frac{{{e^{{\beta _{0k}}+{\beta _{1k}}{X_1}+{\beta _{2k}}{X_2}+ \cdots +{\beta _{pk}}{X_p}}}}}{{1+\sum\nolimits_{{j=}}^{{J - 1}} {{e^{{\beta _{0j}}+{\beta _{1j}}{X_1}+{\beta _{2j}}{X_2}+ \cdots +{\beta _{pj}}{X_p}}}} }}$$

### Evaluation of models

To determine the best statistical model, the fit of the models using the measures of goodness-of-fit, namely, likelihood ratio test, AIC and BIC were compared [27].

#### Likelihood ratio test

The likelihood ratio test (LRT) is a statistical test used for comparing the goodness of fit between two nested models. The test assesses whether the more complex model significantly improves the fit compared to the simpler model [44].

Mathematically, Let ${M_0}$be the simpler or null model with *k* parameters ${\theta _0},{\theta _1}, \ldots,{\theta _k}$ and ${M_1}$be the complex or alternative model with *p* parameters ${\theta _0},{\theta _1}, \ldots,{\theta _p}$, where $p>k$. Then, the likelihood ratio test statistic ($\Lambda$) is defined as the ratio of the likelihood of the data under alternative model to the likelihood of the data under null mode as given in Eq. (6):

6 $$\Lambda =\frac{{{\text{maximized likehood under }}{M_1}}}{{{\text{maximized likehood under }}{M_0}}},$$

where, under ${H_0}$, $\Lambda$ follows chi-square distribution with degrees of freedom $df=p - k$. Therefore,

- If $$\Lambda >\chi _{{\alpha,df}}^{2}$$, reject $${H_0}$$
- If $\Lambda \leqslant \chi _{{\alpha,df}}^{2}$, fail to reject ${H_0}$,

where, $\chi _{{\alpha,df}}^{2}$ is critical value at α level of significant,

#### Akaike information criteria (AIC)

The Akaike information criteria (AIC) is a statistical measure used for comparing the goodness of fit of a model relative to other models. If *k* is the number of estimated parameters in the model, AIC is obtained using Eq. (7) as given below [25]:

7 $${\text{AIC}}= - 2\left( {{\text{log-like}}} \right)+2k$$

The goal is to find the model with the lowest AIC value among the candidate models. A lower value of AIC indicates that the model fits the data better. Compared to the log-likelihood, this criterion takes into consideration the parsimony of the model as it includes a penalty term that increases the number of parameters.

#### Bayesian information criteria (BIC)

The Bayesian information criteria (BIC) criterion, also known as the Schwarz Information Criterion (SIC), is another statistical measure for model selection. The BIC is obtained using Eq. (8) as given below [25]:

8 $${\text{BIC}}= - 2\left( {{\text{log-like}}} \right)+k\log (n)$$

Similar to AIC, BIC helps in evaluating the goodness of fit of different models. A lower value of BIC indicates that the model fits the data better. However, BIC provides a stronger penalty than AIC for additional parameters, which generally makes conservative than AIC in selecting models.

## Results

A total of 1792 T2DM patients were included in the study. The proportion of patients with NLEA, PLEA, and MLEA were 24.9%, 54.0%, and 21.1%, respectively, during hospitalization as presented in Table 1.

**Table 1:** LEA status during study period (*n* = 1792)

Number of LEA	Number	Percentage
NLEA	446	24.9
PLEA	967	54.0
MLEA	379	21.1

Table 2 presents the demographic and clinical variables observed in this study and their distributions across different LEA status. The average age of the patients was 58 years (± 10.7), where most of the patients were between 40 and 60 years old (81.6%). There were more males (51.8%), predominantly I-Taukei (indigenous Fijians) (70%), patients were mostly from rural areas (58.7%). About 15% of the T2DM patients had foot ulcers for one month or longer before presentation at the hospital. Upon presentation to the tertiary centers, most of the patients were afebrile (92.7%). Overall, the median length of hospital stay was five days (IQR 3–7). About 35.4% of the patients were smokers and 42.6% of patients consumed alcohol, 54% of the patients had hypertension, 82.7% of the patients were anemic, approximately 47% of the patients had renal problems, and about 10% of the patient had ischemic heart disease. About 75% of our patients had a higher grade of ulcer (Wagner grade ≥ 4). Most of the patients had uncontrolled blood sugar at admission (75.1%). About 66% of patients had Leukocytosis and a quarter of patients had thrombocytosis (25.5%) upon presentation **(**Table 2**)**.

**Table 2:** Demographic and clinical characteristics among the study population: 2016 to 2019

Factors	Total	Multiple LEA *n* (%)	Primary LEA *n* (%)	No LEA *n* (%)	*P* value
**Total**	1792	379 (21.1)	967(54.0)	446 (24.9)	
**Demographic**					
**Age (years)**, mean ± SD	58.0 ± 10.7	59.0 ± 10.4	58.6 ± 10.8	56.0 ± 11.5	**< 0.001**
**Gender**					**0.077**
Male	928(51.8)	197 (52.0)	520 (53.8)	211 (47.3)	
Female	864(48.2)	182 (48.0)	447(46.2)	235 (52.7)	
**Ethnicity**					**0.016**
I-Taukei	1255(70.0)	278 (73.4)	693(71.7)	284 (63.7)	
FOID*	508(28.3)	94 (24.8)	261(27.0)	153 (34.3)	
Other	29(1.6)	7 (1.8)	13(1.3)	9 (2.0)	
**Location**					0.170
Rural	1052(58.7)	236 (22.4)	571(54.3)	245 (23.3)	
Peri-Urban	269(15.0)	58 (21.6)	136(50.6)	75 (27.9)	
Urban	471(26.3)	85 (18.0)	260(55.2)	126 (26.8)	
**History and Examination**					
**Illness duration**					**< 0.001**
< 1 month	1525(85.1)	313 (82.6)	800 (82.7)	412 (92.0)	
≥ 1 months	267(14.9)	66 (17.4)	167 (17.3)	34 (7.6)	
**Fever**					0.613
Yes	131(7.3)	28 (7.4)	66 (6.8)	37 (8.3)	
No	1661(92.7)	351 (92.6)	901(93.2)	409 (91.7)	
LOS* (days) median (IQR)	5(3,7)	5(3,8)	5 (3,8)	4 (3,6)	< 0.001*
**Wagner**					**< 0.001**
Grade 2	139 (7.8)	0	0	138 (30.9)	
Grade 3	308 (17.2)	0	0	308(69.1)	
Grade 4	608(33.9)	129 (34.0)	479 (59.5)	0	
Grade 5	737(41.1)	250 (66.0)	488 (50.5)	0	
**Lifestyle**					
**Smoking**					**0.046**
Yes	635(35.4)	127 (33.5)	367 (38.0)	141 (31.6)	
No	1157(64.6)	252 (66.5)	600 (62.0)	305 (68.4)	
**Alcohol use**					0.300
Yes	763(42.6)	160 (42.2)	426(44.1)	177 (39.7)	
No	1029(57.4)	219 (57.8)	541(55.9)	269 (60.3)	
**Comorbidities**					
**Hypertension**					**0.006**
Yes	763(53.9)	230 (60.7)	513 (53.1)	222 (49.8)	
No	1029(46.1)	149 (39.3)	454 (46.9)	224 (50.2)	
**Anemia**					**< 0.001**
Yes	965(82.7)	341 (90.0)	815 (84.3)	326 (73.1)	
No	827(17.3)	38 (10.0)	152 (15.7)	120 (26.9)	
CKD*					**0.001**
Yes	1482(46.7)	198 (52.2)	461 (47.7)	178 (39.9)	
No	310(53.3)	181 (47.8)	506 (52.3)	268 (60.1)	
**IHD**					
Yes	186(10.4)	32(8.4)	106 (11.0)	48 (10.8)	0.377
No	1606(89.6)	347(91.6)	861(89.0)	398 (89.2)	
**Stroke**					0.583
Yes	60(3.4)	15 (4.0)	34(3.5)	12(2.7)	
No	1731(96.6)	364 (96.0)	933(96.5)	434(97.3)	
**MI**					0.713
Yes	42(2.3)	7(1.8)	25(2.6)	10(2.2)	
No	1750(97.7)	372(98.2)	942(97.4)	436(97.8)	
CABG*					0.110
Yes	8(0.4)	4(1.1)	2(0.2)	2(0.4)	
No	1784(99.6)	375(98.9)	965(99.8)	444(99.6)	
**Investigations**					
**Uncontrolled Blood Sugar**					0.368
Yes	1345(75.1)	276 (72.8)	738(76.3)	331 (74.2)	
No	447(24.9)	103 (27.2)	229(23.7)	115 (25.8)	
**Leukocytosis**					**< 0.001**
Yes	1179(65.8)	279 (73.6)	651(67.3)	249 (55.8)	
No	613(34.2)	100 (26.4)	316(32.7)	197(44.2)	
**Thrombocytosis**					
Yes	457(25.5)	129(34.0)	249(25.7)	79(17.7)	**< 0.001**
No	1335(74.5)	250(66.0)	718(74.3)	367(82.3)	

Values are presented as number (%) or mean ± SD or median (IQR). *Used non-parametric methods. *FOID: Fijians of Indian Decent, *CABG: coronary artery bypass grafting, *LOS: Length of stay, *IHD: Ischemic Heart Disease, *CKD: Chronic kidney disease, *MI: Myocardial Infarction

Before developing the model, bivariate analysis was carried out to determine if significant associations exited between factors (predictor variables) and LEA status (outcome variable) as well as to identify empty or extremely small cells for categorical data. Usually the model may become unstable, or it might not run at all due to empty cells. Table 2 shows that, out of 20 available variables, 11 variables were statistically significant which includes age, ethnicity, illness duration, length of stay, Wagner grade, smoking, hypertension, anemia, renal problem, leukocytosis, and thrombocytosis. These factors showed a significant association with the outcome variable.

### Proportional odds model

Proportional Odds model (POM) was performed to find out the potential the risk factors of MLEA. Since the outcome variable has three ordered categories, two logits were modeled such as (1) NLEA versus PLEA and MLEA; and (2) NLEA and PLEA versus MLEA. The results of POM are presented in Table 3, which includes the cutoff thresholds, estimated coefficients of the predictors, associated standard errors (SE), the p-value and the odds ratio with 95% confidence intervals. The *polr* command from the *MASS* package in R software was employed to fit the POM.

**Table 3:** Factors associated with LEA using proportional odds model

Variables	Coefficient	SE	*p*-value	OR (95% Cls)	Single Brant test *p*-value
**Age (in years)**	0.01	0.01	**0.002**	1.01 (1.01, 1.02)	0.10
**Gender**					
Female				1	
Male	0.25	0.09	**0.008**	1.28 (1.07, 1.54)	**0.03**
**Ethnicity**					
I-Taukei				1	
FOID	-0.29	0.10	**0.004**	0.74 (0.61, 0.91)	0.36
Other	-0.09	0.37	0.795	0.91 (0.44, 1.88)	0.31
**Illness duration**					
<1 month				1	
≥ 1 months	0.49	0.13	**< 0.001**	1.64 (1.28, 2.11)	**0.00**
**Length of Stay**	0.05	0.01	**< 0.001**	1.05 (1.03, 1.07)	**0.00**
**Hypertension**					
No				1	
Yes	0.32	0.09	**0.001**	1.38 (1.15, 1.66)	0.32
**Anemia**					
No				1	
Yes	0.60	0.13	**< 0.001**	1.83 (1.43, 2.34)	0.76
**CKD**					
No				1	
Yes	0.17	0.09	0.085	1.18 (0.98, 1.13)	0.66
**Leukocytosis**					
No				1	
Yes	0.44	0.10	**< 0.001**	1.56 (1.28, 1.89)	0.66
**Thrombocytosis**					
No				1	
Yes	0.47	0.10	**< 0.001**	1.61(1.30, 1.98)	0.84
Intercept 1	1.20	0.29	-	-	
Intercept 2	3.81	0.31	-	-	

Results in Table 3 showed age, gender, ethnicity, illness duration, length of stay, hypertension, anemia, leukocytosis, and thrombocytosis were strongly significant risk factors at 5% level of significance, while the effect of renal problem was not significant (*p* = 0.085).

In POM, a positive coefficient indicates that a unit increase in the independent variable increases the likelihood of occurrence of MLEA and a negative coefficient indicates that a unit increase in the independent variable decreases the likelihood of occurrence of MLEA. For example, the probability of occurrence of MLEA is higher among males compared to females (OR = 1.28), while keeping other predictors constant. Before interpreting the odds ratio, we need check whether PO assumption for POM is valid or not.

If the assumption does not hold the interpretation of the odds ratio for the independent variables may be invalid. The intercept 1 and intercept 2 values in Table 3 are the threshold cut off values that separate different LEA status. The intercept 1 in Table 3 indicates that 1.20 is the estimated cut-off point on the response variable used to differentiate NLEA from PLEA and MLEA when all predictors are zero. Similarly, intercept 2 (3.81) is the estimated cut-off point to differentiate NLEA and PLEA from MLEA. These thresholds reflect the predicted cumulative probabilities when the independent variables take the zero value. Since the confidence intervals for the two thresholds do not overlap which indicate the three different LEA status categories are significantly different from each other.

The Proportional Odds (PO) assumption was tested using Brant test implemented in *brant* package in R. This test was used to check whether PO assumption is violated or not. To identify which predictor variables violated the assumption, a single Brant test for each predictor variables were examined. An insignificant test statistic provides evidence that the parallel regression assumption has been violated. Overall, the Brant test yielded a Chi-square value of 35.34 with p-value of 0.00, which suggests that the PO assumption has been violated. The p-values of the single Brant tests are shown in the last column of Table 3. The results show that the parallel lines assumption among different LEA categories is violated for only three variables gender (*p* = 0.03), los (*p* = 0.00), illness duration (*p* = 0.00). However, all other seven variables (age, ethnicity, hypertension, anemia, renal problem, leukocytosis, and thrombocytosis) satisfied the assumption. Since overall as well as some individual predictors do not hold the assumption, the POM seems to be not appropriate. Thus, there was a need to fit a partial proportional odds model (PPOM) to remodel the data. Since the formulation of the logit functions are same in POM and PPOM, these two models are comparable [37].

### Partial proportional odds model

A PPOM was fitted because the data violated the assumption. Since the outcome variable has three ordered categories, the results present two sets of models. The first model (Model I) is a binary logistic regression model where the dependent variable is considered as NLEA vs. PLEA & MLEA. The second model (Model 2) dependent variable is considered as NLEA &. PLEA vs. MLEA, which is the focus in this study. As shown in Table 4, PPOM presented two models and two set of results, which includes the estimated coefficients of the predictors, associated SE, the odds ratio with 95% confidence intervals, and the p-value for each predictor in each model.

A restricted partial proportional odds model (PPOM-R) was fitted to the data using the *vglm()* function from the *VGAM* R package [45], where the parallel-lines constraint was relaxed only for those variables where the assumption was not justified thus the parallel-lines constraint was considered for the other variables to satisfy the assumption [45]. Thus, the predictors that met the proportional odds assumption (i.e. age, ethnicity, hypertension, anemia, renal, leukocytosis, and thrombocytosis) have the same coefficient values in the two models in Table 4.

**Table 4:** Results of partial proportional odds model (PPOM)

Variable	Model 1				Model 2			
	Coefficient	SE	OR (95% Cls)	*p*-value	Coefficient	SE	OR (95% Cls)	*p*-value
**Intercept**	-1.58	0.30			-3.46	0.31		
**Age**	0.01	0.01	1.01 (1.01, 1.02)	**0.001**	0.01	0.01	1.01 (1.01, 1.02)	**0.001**
**Gender**								
Female			1				1	
Male	0.40	0.12	1.50 (1.19, 1.88)	**< 0.001**	0.09	0.12	1.10 (0.87, 1.39)	0.398
**Ethnicity**								
IT			1				1	
FOID	-0.29	0.10	0.75 (0.61, 0.91)	**0.005**	-0.29	0.10	0.75 (0.61, 0.91)	**0.005**
Other	-0.10	0.36	0.91 (0.44, 1.85)	0.782	-0.10	0.36	0.91 (0.44, 1.85)	0.782
**Illness duration**								
< 1 month			1				1	
≥ 1 months	0.93	0.19	2.53 (1.72, 3.73)	**< 0.001**	0.23	0.16	1.26 (0.92, 1.72)	0.150
**LoS**	0.10	0.02	1.10 (1.07, 1.14)	**< 0.001**	0.01	0.01	1.01 (0.99, 1.04)	0.301
**Hypertension**								
No			1				1	
Yes	0.31	0.09	1.37 (1.14, 1.64)	**0.002**	0.31	0.09	1.37 (1.14, 1.64)	**0.002**
**Anemia**								
No			1				1	
Yes	0.59	0.13	1.80 (1.41, 2.31)	**< 0.001**	0.59	0.13	1.80 (1.41, 2.31)	**< 0.001**
**CKD**								
No			1				1	
Yes	0.16	0.10	1.17 (0.97, 1.41)	0.104	0.16	0.10	1.17 (0.97, 1.41)	0.104
**Leukocytosis**								
No			1				1	
Yes	0.45	0.10	1.56 (1.28, 1.90)	**< 0.001**	0.45	0.10	1.56 (1.28, 1.90)	**< 0.001**
**Thrombocytes**								
No			1				1	
Yes	0.47	0.10	1.60 (1.30, 1.98)	**< 0.001**	0.47	0.10	1.60 (1.30, 1.98)	**< 0.001**

In PPOM, the values of the coefficients and odds ratios for covariates were found slightly different compared to POM. Moreover, all the variables that were significant in POM are significant in Model 1, but some of the variables were found to be statistically insignificant in Model 2.

Based on Model 2 in Table 4 as per as the interest in this research, the results showed only age, ethnicity, hypertension, anemia, leukocytosis, thrombocytosis were identified as risk factors of MLEA. The coefficients can be interpreted in the same way as predictors in POM. A positive coefficient indicates that a unit increase in the independent variable increases the likelihood of occurrence of MLEA and a negative coefficient indicates that a unit increase in the independent variable decreases the likelihood of occurrence of MLEA. For example, based on PPOM there was 1.01 times increased risk of having MLEA for every one-year increase (OR:10.1 95% CI 1.01, 1.02). The odds of having MLEA decreased by 25% in FOID as compared to I-Taukei (OR:0.75, 95% CI 0.61, 0.91). All these effects or relationships stated herein are subject to the ceteris paribus assumption of keeping all other predictors in the model constant while interpreting the effects of a predictor.

### Multinomial logistic regression model

Multinomial Logistic Regression (MLR) model, another analysis tool that can be used as an alternative of POM when the PO assumption violates [43]. In this study, MLR model was developed due to violation of the assumption. NLEA was treated as the reference category, thus there is no model for NLEA. The MLR model estimated two sets of binary logit models: NLEA vs. PLEA, NLEA vs. MLEA. Therefore, we obtain different coefficients for the predictors in the two models. The results of MLR are presented in Table 5, which includes the estimated coefficients, SE, the p-value and the odds ratio with 95% confidence intervals for each predictor in each model. The predictors with positive coefficients corresponding to LEA status indicate an increase in the probability of occurrence of LEA or MLEA compared to the reference category. MLR model was fitted to the data using *multinom ()* function from the *nnet* R package.

**Table 5:** Factors associated with LEA or MLEA by MLR model

Variable	PLEA				MLEA				
	Coefficient	SE	OR (95% Cls)	*p*-value	Coefficient	SE	OR (95% Cls)	*p*-value	
**Intercept**	-1.865	0.36		**< 0.001**	-3.80	0.48		**< 0.001**	
A**ge**	0.018	0.01	1.02 (1.01,1.03)	**0.001**	0.021	0.01	1.02 (1.01, 1.04)	**0.004**	
**Gender**									
Female			1				1		
Male	0.408	0.12	1.50 (1.18, 1.91)	**0.001**	0.399	0.15	1.49 (1.11, 2.00)	**0.008**	
**Ethnicity**									
IT			1				1		
FOID	− 0.348	0.13	0.71 (0.55, 0.91)	**0.007**	-0.480	0.16	0.62 (0.45, 0.85)	**0.003**	
Other	− 0.444	0.46	0.64 (0.26, 1.56)	0.329	-0.167	0.54	0.85 (0.29, 2.44)	0.757	
**Illness duration**									
< 1 month			1				1		
≥ 1 months	0.914	0.20	2.49 (1.68, 3.71)	**< 0.001**	0.934	0.23	2.54 (1.62, 4.01)	**< 0.001**	
**LoS**	0.100	0.02	1.11 (1.07, 1.15)	**< 0.001**	0.094	0.02	1.10 (1.06, 1.15)	**< 0.001**	
**Hypertension**									
No			1				1		
Yes	0.161	0.12	1.17 (0.93, 1.49)	0.186	0.508	0.15	1.66 (1.24, 2.23)	**< 0.001**	
**Anemia**									
No			1				1		
Yes	0.453	0.15	1.57 (1.18, 2.10)	**0.002**	0.907	0.21	2.48 (1.64,3.75)	**< 0.001**	
**Leukocytosis**									
No			1				1		
Yes	0.392	0.13	1.48 (1.16, 1.89)	**0.002**	0.669	0.16	1.95 (1.43, 2.67)	**< 0.001**	
**Thrombocytes**									
No			1				1		
Yes	0.387	0.15	1.47 (1.10, 1.98)	**0.010**	0.743	0.17	2.10 (1.50, 2.95)	**< 0.001**	
**CKD**									
No			1				1		
Yes	0.171	0.13	1.19 (0.93, 1.52)	0.178	0.246	0.15	1.28 (0.95, 1.73)	0.111	

The covariance significance levels were not significantly different for the two categories of LEA status. However, more variables were found to be significant for the MLEA compared to the PLEA (9 vs. 8 respectively). The predictors with positive coefficients corresponding to a LEA status indicate an increase in the probability of occurrence of PLEA or MLEA compared to the reference category, and vice versa. For example, the odds ratios estimated for the variable gender for PLEA and MLEA with NLEA were 1.50, and 1.49, respectively. This indicates that the odds of having PLEA compared to NLEA is 50% higher for males compared to Females. Similarly, the odds of having MLEA compared to NLEA is 49% higher for males compared to Females. The POM cannot provide this important information because of the proportional odds assumption. In POM, only shows that the odds of having MLEA is 1.28 times higher in males compared to females (Table 3).

### Comparison of the developed models POM, PPOM and MLR

In the present study, as discussed earlier, the POM appears to be an inappropriate model since the overall model and some of the predictor variables did violate the PO assumption. Accordingly, two alternative models (i.e. PPOM and MLR) were fitted and compared with POM. The statistical measures AIC and BIC were used to evaluate the choice among three models. Smaller values of these statistics indicate a better model fitted for the data. The results of the comparison measures are presented in Table 6, which shows that PPOM has lowest value of AIC and BIC indicating better performance of the model compared to the other two models. Moreover, likelihood ratio (LR) test was also used to evaluate the choice between three models and the results of the test are presented in table, which shows that PPOM has higher value of LR compared to the other two models. Thus, suggested PPOM is the preferred model (*p* < 0.0001).

**Table 6:** Comparison of the developed models

Model	AIC	BIC	LR
POM	3472.94	3544.33	-1723.47
PPOM	3449.34	3531.71	-1703.99
MLR	3457.62	3589.40	-1704.81

Moreover, the performance analysis of PPOM is carried out using calibration plot and, ordinal c-statistic and Somers’ D statistics.

**Fig. 2:**
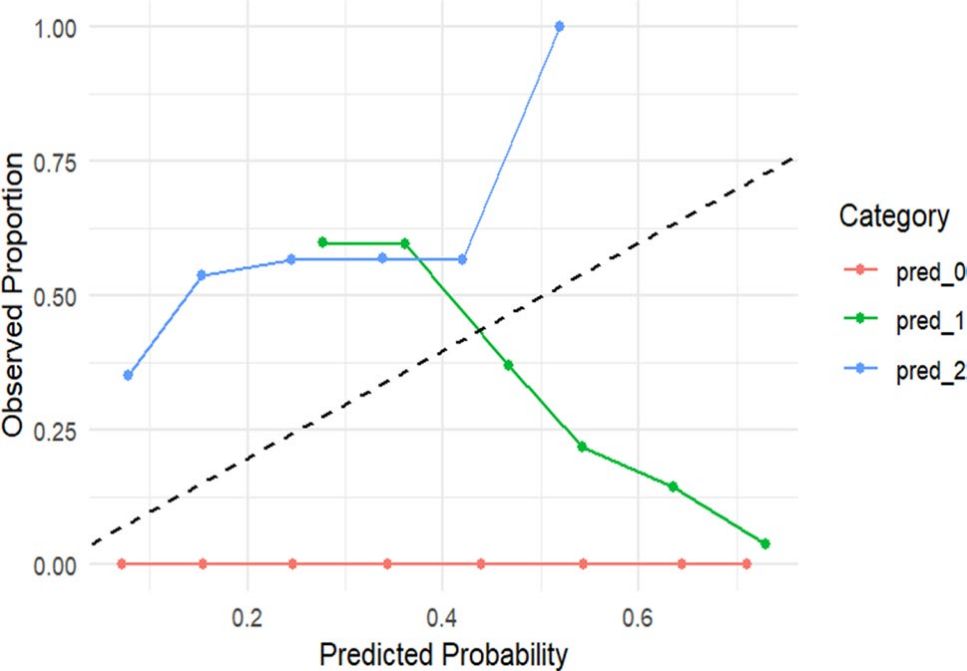
Calibration Plot

The calibration plot (Fig. 2) for the PPOM showed variable performance across outcome categories. Predicted probabilities for No LEA remained flat, indicating poor calibration. For Primary LEA, the model over-predicted at lower probabilities and under-predicted at higher levels, crossing the ideal line at 0.45. Multiple LEAs were consistently over-predicted at higher risks. While the model reasonably differentiates risk categories, absolute predicted probabilities for No LEA and Multiple LEAs are less reliable, warranting future recalibration. Moreover, the PPOM demonstrated acceptable discrimination, with an ordinal c-index of 0.70 and a corresponding Somers’ D of 0.40, indicating moderate concordance between predicted risks and observed outcomes. This suggests the model correctly ranks patients by risk about 70% of the time, supporting its potential for clinical risk stratification.

## Discussion

With the outcome variable being ordinal, an initial POM was fitted at first using 10 suitable predictors. Upon testing for the PO assumption, this model appeared not to be an appropriate choice of model due to the violation of the proportionality assumption. On the other hand, the fitting of the MLR model does not require the outcome to satisfy the proportional odds assumption. However, the MLR models do not account for the hierarchical nature of the LEA status. Thus, the PPOM model was fitted as a solution to this problem and was considered a better and more appropriate model compared to POM.

As shown in Tables 3 and 4, the coefficients and odds ratios for the predictors differ slightly between the PPOM and POM models. Notably, all variables significant in POM remain significant in Model 1 of PPOM. However, some variables become insignificant in Model 2 of PPOM. Several predictors exhibited non-proportionality, meaning their effects differed across outcome thresholds. As a result, some variables were significant in the POM, which allows effects to vary where the PO assumption is violated. For example, certain factors may increase the likelihood of developing any LEA compared to no LEA but have minimal influence on the progression from PLEA to MLEA. These differences highlight the importance of using a PPOM, which provides more flexible, threshold-specific estimation of predictor effects. Moreover, a smaller number of predictors are statistically significant in Model 2 compared to the POM (6 vs. 9).

The PPOM results identified several significant predictors of MLEA, encompassing both demographic and clinical variables. Demographic variables such as age, gender, and ethnicity were important, with older patients, males, and those from the I-Taukei ethnic background facing a higher likelihood of MLEA. Additionally, clinical factors including a history of comorbidities such as hypertension, anemia, leukocytosis, thrombocytosis, and prolonged ulcer duration prior to admission were strongly associated with an increased risk of MLEA in patients with T2DM. These findings align with previous research [5, 8, 46–55], highlighting the complex interplay of demographic and clinical determinants in the progression to MLEA.

### Clinical implications and potential interventions

The identification of hypertension, anemia, leukocytosis, and prolonged ulcer duration as significant predictors of MLEA has important clinical implications. These factors can serve as critical indicators in routine diabetic foot assessments and should prompt intensified monitoring and early multidisciplinary intervention by the healthcare professionals. For example, the presence of anemia or thrombocytosis may reflect systemic inflammation or poor wound healing capacity, warranting hematological evaluation and correction. Likewise, patients presenting with prolonged duration prior to admission may benefit from fast-track referral pathways to specialized foot care clinics. Proactive management of hypertension and leukocytosis could also mitigate the risk of vascular complications and infection related deterioration. Collectively, these insights support the development of clinical risk scores and tailored care pathways to identify high-risk individuals and reduce the incidence of MLEA in T2DM populations.

### Strengths and limitations

Published data on diabetes and LEA in Fiji is limited. This study provides valuable evidence on LEA among T2DM patients and is the first to examine risk factors of MLEA in Fiji. The findings fill key gaps in literature and highlight the role of demographics and clinical factors in amputation risk.

This study has several limitations. Firstly, its retrospective design introduces potential biases, such as reliance on the accuracy and completeness of existing medical records, which may result in missing or inconsistent data. Several clinically important variables such as the duration of diabetes, glycosylated hemoglobin (HbA1c), lipid profiles, and neurovascular status were excluded due to inadequate documentation, potentially affecting the validity and reliability of the findings. Additionally, unmeasured confounding may be present, as several potentially important factors known to influence MLEA risk were not available in the medical records, including socioeconomic status, medication adherence, and others. Furthermore, the study’s scope is restricted to tertiary hospitals in Fiji, which may limit the generalizability of the results to other healthcare settings, such as primary or secondary care facilities, or to different geographic or socio-economic contexts within or beyond Fiji. Therefore, caution should be exercised when extrapolating these findings to broader populations or health systems.

The novelty of this research lies in its focus on the ordinal classification of LEA status, addressing a gap in previous studies that typically employed binary outcomes. Our findings demonstrate that PPOM outperformed both the POM and the MLR models in analyzing status, establishing PPOM as an effective approach for modeling LEA status. This research is particularly relevant given Fiji’s alarmingly high prevalence of diabetes and the significant contribution of T2DM to mortality rates, which poses a serious public health challenge. Despite this, there is a notable absence of published data on risk factors for amputations in Fiji, and no current statistics on amputation rates among T2DM patients.

## Conclusion

This study offers a comprehensive analysis of the demographic and clinical characteristics associated with multiple lower extremity amputations (MLEA) diabetic inpatients with foot ulcers in Fiji. By categorizing patients into three groups: NLEA, PLEA, and MLEA, it underscores the varying risk levels for limb loss within this population. Recognizing the ordinal nature of LEA, we introduced a Partial Proportional Odds Model (PPOM) to identify associated risk factors among patients with T2DM.

To our knowledge, this is the first study in Fiji to address these knowledge gaps, offering insights into the complex interplay of risk factors contributing to amputations among T2DM patients. It highlights the importance of considering both demographic and clinical factors in understanding and mitigating LEA risk. Future research should aim to refine predictive models and develop targeted interventions to reduce LEA risk, ultimately improving outcomes in diabetes management in Fiji. The impact of this research lies not only in its methodological innovation but also in its potential to inform clinical decision-making and public health strategies. By highlighting the urgent need for targeted interventions and improved diabetes management, this work has the potential to reduce the incidence of amputations and improve patient outcomes in Fiji and similar high-risk populations globally.

## Electronic supplementary material

Below is the link to the electronic supplementary material.

Supplementary Material 1

Supplementary Material 2

## Data Availability

Data can be made available on request to the corresponding author subject to approval from the relevant organization.

## Abbreviations

AIC: Akaike’s Information Criterion
BIC: Bayesian Information Criterion
CABG: Coronary artery bypass grafting
CKD: Chronic Kidney Disease
CRM: Continuous Ratio Model
FOID: Fijians of Indian Decent
ICD: International Classification of Diseases
LEA: Lower Extremity Amputation
LR: Likelihood Ratio
LOS: Length of stay
MLEA: Multiple Lower Extremity Amputation
NHN: National Health Number
POM: Proportional Odds Model
PPOM: Partial Proportional Odds Model
PLEA: Primary Lower Extremity Amputation
PATIS: Patient Information System
SM: Stereotype Model
T2DM: Type 2 Diabetes Mellitus
